# Tissue specific alpha-2-Macroglobulin (A2M) splice isoform diversity in Hilsa shad, *Tenualosa ilisha* (Hamilton, 1822)

**DOI:** 10.1371/journal.pone.0216144

**Published:** 2019-07-23

**Authors:** Vindhya Mohindra, Tanushree Dangi, Labrechai Mog Chowdhury, J. K. Jena

**Affiliations:** 1 ICAR-National Bureau of Fish Genetic Resources (ICAR-NBFGR), Lucknow, India; 2 Indian Council of Agricultural Research (ICAR), Krishi Anusandhan Bhawan—II, New Delhi, India; Shanghai Ocean University, CHINA

## Abstract

The present study, for the first time, reported twelve A2M isoforms in *Tenualosa ilisha*, through SMRT sequencing. Hilsa shad, *T*. *ilisha*, an anadromous fish, faces environmental stresses and is thus prone to diseases. Here, expression profiles of different A2M isoforms in four tissues were studied in *T*. *ilisha*, for the tissue specific diversity of A2M. Large scale high quality full length transcripts (>0.99% accuracy) were obtained from liver, ovary, testes and gill transcriptomes, through Iso-sequencing on PacBio *RSII*. A total of 12 isoforms, with complete putatative proteins, were detected in three tissues (7 isoforms in liver, 4 in ovary and 1 in testes). Complete structure of A2M mRNA was predicted from these isoforms, containing 4680 bp sequence, 35 exons and 1508 amino acids. With *Homo sapiens* A2M as reference, six functional domains (A2M_N,A2M_N2, A2M, Thiol-ester_cl, Complement and Receptor domain), along with a bait region, were predicted in A2M consensus protein. A total of 35 splice sites were identified in *T*. *ilisha* A2M consensus transcript, with highest frequency (55.7%) of GT-AG splice sites, as compared to that of *Homo sapiens*. Liver showed longest isoform (X1) consisting of all domains, while smallest (X10) was found in ovary with one Receptor domain. Present study predicted five putative markers (I-212, I-269, A-472, S-567 and Y-906) for EUS disease resistance in A2M protein, which were present in MG2 domains (A2M_N and A2M_N2), by comparing with that of resistant and susceptible/unknown response species. These markers classified fishes into two groups, resistant and susceptible response. Potential markers, predicted in *T*. *ilisha*, placed it to be EUS susceptible category. Putative markers reported in A2M protein may serve as molecular markers in diagnosis of EUS disease resistance/susceptibility in fishes and may have a potential for inclusion in the marker panel for pilot studies. Further, challenging studies are required to confirm the role of particular A2M isoforms and markers identified in immune protection against EUS disease.

## Introduction

The alpha-2-Macroglobulin (A2M) is a broad-spectrum protease-binding protein and evolutionarily conserved component of the innate immune system in vertebrates [1]. It provides protection against invading pathogens by trapping and inhibiting all classes of microbial and parasitic proteases [1]. The protective role of this non-specific protease inhibitor has been studied in various bacterial, viral and fungal diseases affecting aquaculture [2,3]. The expression of A2M in defence against pathogens has been reported in number of fish species i.e. grass carp [4], rainbow trout and brook charr [5], common carp [6], gilthead seabream [7], plaice [8] and brook trout [9].

Alternative splicing (AS) of multiple exons is a major source of transcripts and leads to isoform and proteome diversity in eukaryotic organisms [10,11] and better understanding of the disease response requires the knowledge of full complement of mRNA isoforms [12,13]. Previous studies have used conventional methods of cloning and sequencing for identification of A2M isoforms in aquatic animals i.e. common carp [14,6]and Chinese shrimp [15] against microbial infection. However, the recent isoform sequencing (Iso-Seq) based on Single Molecule Real Time (SMRT) technology offers the rapid identification of AS events accurately [16], where entire RNA molecules can be sequenced without any fragmentation or post-sequencing assembly.

*Tenualosa ilisha*, commonly known as Hilsa, is an economically important anadromous fish species, which migrates from marine to freshwater for sexual maturity [17] and faces different environmental fluctuations, including salinity and temperature gradients during its life span as well as pollution levels in the river systems, make them more prone to diseases. There are reports of bacterial [18] and protozoan parasites infections [19] in wild Hilsa, however, there is no information in Hilsa about the Epizootic ulcerative syndrome (EUS) in wild.

EUS is one of the major diseases of aquaculture caused by oomycetes fungus, *Aphanomyces invadans*, which affects farmed and wild fishes, worldwide [20]. Little is known about EUS susceptibility in many fish species in wild populations [21]. This disease is recognized as reportable disease due to its broad host susceptibility nature and potential for further spread [22]. Majority of fishes have been reported to be susceptible for EUS [23,24], while only few like *Cyprinus carpio* [25] and *Oreochromis niloticus* [26] have been recognized as resistant species. To understand the species specific susceptibility/resistance to EUS, Yadav et al [27] reported the significant decrease in activity of innate immune components as ά-A2M, anti-proteases and lysozyme in advance stage of *A*. *invadans* infection in susceptible *Labeo rohita;* while no significant modulation was observed in resistant *C*. *carpio* [25]. McTaggart et al [28] hypothesised that small number of genes in immune system show positive selection signatures, as a result of host-parasite interactions. In four plant species, disease resistance signature in the leucine-rich repeat receptor-like kinase genes was reported [29]. It is proposed in the present study that A2M can be a candidate gene for finding out disease resistance signatures to EUS disease in fish species.

In the present study, full length isoform sequencing was employed to characterize the tissue specific potential isoforms of A2M gene in Hilsa shad, *Tenulosa ilisha* and using these isoforms, a complete structure of A2M gene was predicted. Attempts have also been made to identify the signatures of disease resistance in A2M gene to the dreaded disease, EUS, in several fish species, on the basis of A2M protein sequences.

## Materials & methods

### Ethical statement

The following protocols followed were approved by Institutional Animal Ethical Committee (IAEC), ICAR-NBFGR, Lucknow, India vide No. G/CPCSEA/IAEC/2015/2 dated 27 Oct., 2015.

### Sample collection

Adult *T*. *ilisha* fish were collected from commercial catches, at the site of collection from natural fresh water habitat (Padama River; N 24^o^ 80’, E 87^o^ 93’, Farrakka, West Bengal, India) and euthanized with MS222 (Sigma Aldrich, USA). The tissue samples, liver, ovary testes and gill were dissected out and snap frozen in liquid N_2,_ transported to laboratory in frozen condition and transferred to -80°C, till analysis.

### Transcriptome sequencing and analysis

Total RNA was isolated from four frozen tissues (Liver, Ovary, Testes and Gill) using guanidinium thiocyanate-phenol-chloroform extraction method (Trizol) method followed by purification with nucleic acid extraction kit (Nucleo Spin RNAII, Germany) and purity of RNA was assessed on DS-11 Nanodrop spectrophotometer (Denovix, DeNovix Inc., US). For double stranded cDNA synthesis and Iso-Seq cDNA library preparation, PacBio Isoform-sequencing protocol was followed (http://www.pacb.com/support/documentation). Long read Single-molecule Real Time sequencing was performed on the PacBio RSII using P6-C4 chemistry. Raw reads obtained were processed using the RS_IsoSeq pipeline in Pacific Biosciences' SMRT analysis software version 2.3.0 (https://github.com/PacificBiosciences/SMRT-Analysis) to classify full length and non-full length isoforms. High quality consensus (Minimum Quiver Accuracy > = 0.99) were obtained by polishing full length reads with Quiver algorithm [30].

### Identification of alpha-2-Macroglobulin-Like (A2ML)Isoforms and splice junction

To identify immune genes from all transcriptomes, high quality transcripts were analyzed through KAAS server [31] for pathway annotations. A2ML isoforms were screened from immune genes after removing redundant sequences by CD-HIT Suite (http://weizhong-lab.ucsd.edu/cdhit_suite/cgi-bin/index.cgi?cmd=cd-hit). The A2M1 transcript sequence of *Homo sapiens* (XM_017018870.1; https://www.ncbi.nlm.nih.gov/ieb/research/acembly) was used as a reference for comparative analysis. Non-redundant set of A2M isoforms (nucleotide sequences) were aligned in ClustalW (www.genome.jp/tools/clustalw/) against *Homo sapiens* A2ML1gene (XM_017018870.1 and NP_000005.2) to decipher exon-intron boundaries and splice variants. The frequency of splice sites was predicted and graphically represented using WebLogo tool (http://weblogo.berkeley.edu/logo.cgi). Hilsa A2M consensus sequence was generated by aligning and computing overlapping reads of seven A2M isoforms using CAP3 program [32] in Bioedit version 7.2.5 [33]. Blastx searches were performed for each isoform against NCBI database, to identify domains and motifs in A2M protein. Gene structure of all A2ML isoforms were drawn using web based tool Exon Intron Graphic Maker (http://wormweb.org/exonintron). To decipher differences in domain regions, multiple sequence alignments of deduced amino acid sequence of Hilsa A2M (consensus) was done with other sequences from GenBank database (*Homo sapiens*: NP_000005.2, *Danio rerio*: NP_001132951.1 and *Clupea harengus*: XP_012689768.1). Functional domains and superfamilies were determined by SMART tool (smart.embl-heidelberg.de) and Superfamily 1.75 (supfam.org/SUPERFAMILY), respectively.

### Characterization of isoforms

To identify complete open reading frame (ORF), isoforms of A2ML transcriptsfrom all tissues were characterized with GenScan tool (http://genes.mit.edu/GENSCAN.html). The Expasy's ProtParam server (http://www.expasy.org) was used to decipher the physicochemical properties of A2ML consensus transcripts, i.e. amino acid composition, molecular weight, theoretical isoelectric point (pI), instability index (II), extinction coefficient (EC), Alipathic Index (AI) and grand average of hydropathicity (GRAVY). Presence of disulfide bonds, phosphorylation sites for serine, threonine and tyrosine in consensus transcripts were identified using DiANNA 1.1 web server (http://clavius.bc.edu/~clotelab/DiANNA) and NetPhos 3.1 (www.cbs.dtu.dk/services/NetPhos). The possible N-glycosylation sites were predicted through NetGlyc 1.0 server (http://www.cbs.dtu.dk/services/NetGlyc). Sub-cellular localization sites of A2ML protein and its potential signal peptides were depicted through web-based tools, PSORT (http://psort.hgc.jp/) and SignalP 4.1 (http://www.Cms.dtu.dk/services/SignalP). To find out the functional domains, *T*. *ilisha* A2ML consensus protein was aligned with that of human (https://www.ncbi.nlm.nih.gov/protein/66932947/; http://www.uniprot.org/uniprot/P01023).

### Identification of putative markers for disease response

To find out putative markers for response to Epizootic ulcerative syndrome **(**EUS) disease in fishes, EUS resistant, susceptible and moderately susceptible species were selected from previous studies [25–27,34–36]. These included A2ML protein sequences of three disease resistant species (*Cyprinus carpio*, *Oreochromis niloticus* and *Maylanadia Zebra)*, six susceptible species (*Fundulus heteroclitus*, *Clupea harengus*, *Ctenopharyngodon idella*, *Salmo salar*, *Carassius gibelio and Esox lucius*) and eight species (*Takifugu rubripes*, *Poecilia reticulate*, *Labrus bergylta*, *Larimichthys crocea*, *Monopterus albus*, *Danio rerio*, *Rachycentron canadum* and *Tenulosa ilisha*, *present study*) with unknown response to disease, which were downloaded from NCBI database (https://www.ncbi.nlm.nih.gov) and aligned in ClustalwPutative differentiating amino acids were identified manually by comparing all sequences in BioEdit [33].

Based on the identified putative markers (amino acids) in 18 fish species, grouping was done on the basis of phylogenetic tree, constructed using Maximum Likelihood method with 1000 bootstrapsin MEGA version 5.0 [37]. The best substitution model Jones-Taylor-Thornton (JTT) was used for amino acids at uniform rates among all sites. The parameters for JTT were lowest BIC scores (Bayesian Information Criterion: 347.306), AICc value (Akaike Information Criterion, corrected: 393.87) and Maximum Likelihood value (*lnL*: -107.835).

## Results

### Identification and characterization of A2ML isoforms in *Tenualosa ilisha*

To study comprehensive isoform pattern in four tissues, a total of 28351, 30696, 17429 and 38232 high quality full length transcripts (>0.99% accuracy) were obtained from Isoseq analysis of liver, ovary, testes and gill transcriptomes. BioSample and SRA accession and amount of data generated are shown in Table A in S1 File. A total of 50, 4 and 1 initial transcripts were found associated to A2ML gene specific to liver, ovary and testes and no isoform among gill transcripts. Finally, a total of 12 isoforms were identified when compared with reference A2ML1 gene structure (*Homo sapiens;* 5229 bp, 35 exons). A total of 7 splice variants in liver, 4 in ovary and 1 in testes, were identified, which encoded putatively complete proteins. In *T*. *ilisha*, the complete consensus mRNA sequence, obtained from these splice variants, contained 4680 bp, with 35 exons and encoded the deduced protein of 1508 amino acids (Figure A in S1 File). Blastx search of *T*. *ilisha* A2ML gene showed highest similarity with *Clupea harengus* (XP_012689768.1), followed by *Cyprinus carpio* (BAA85038.1), *Danio rerio* (NP_001132951), *Ctenopharyngodon idella* alpha-2-macroglobulin (AAR00337) and *Homo sapiens* (NP_000005.2) (Table B in S1 File).

**Fig 1:**
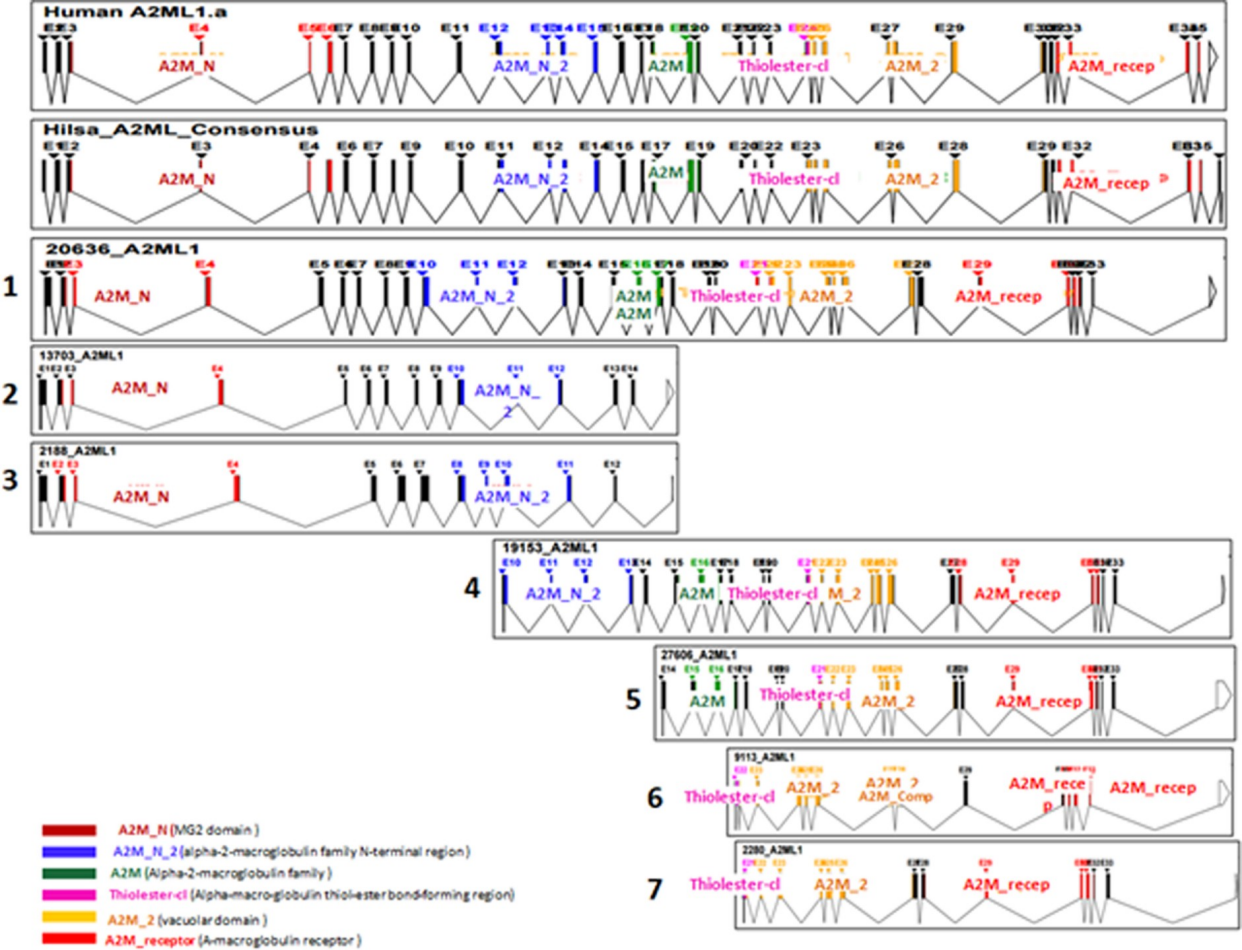
Alternative splice variants of A2ML1 gene (*T*. *ilisha)* showing twelve isoforms from liver (1–7), ovary (8–11) and testes (12). Reference gene is Human A2ML1 isoform representing total six domains (A2M_N, A2M_N2, A2M, Thiolester-cl, Complement and Receptor domain) with total 35 numbers of exons, 5’ UTR (73 bp) and 3’ UTR (359 bp) region.

The longest (IsoformX1) and smallest isoforms (isoformX10) contained 4431 bp sequence (1477 amino acid) and 399 bp (95 amino acid) in their transcripts, respectively. The 12 *T*. *ilisha*A2ML isoforms along with exons, introns and domains are shown in Fig 1 and complete open reading frame (ORF) characteristics in Table 1.

**Table 1:** Characteristics of tissue specific A2ML isoforms in *Tenualosa ilisha*.

Organisms	Isoforms	mRNA variant ID	mRNA length (bp)	Open reading Frame (bp)	Best predicted Protein (aa)	Exons in CDS	5’ UTR (bp)	3’ UTR (bp)
***Homo sapiens*** ^#^	Isoform a	A2ML1.a ^#^	5229	4797	1454	35	73	359
***T*. *ilisha***	Consensus	A2ML	4680	4524	1508	35	47	109
**Liver**	IsoformX1	20636	4431	4128	1477	33	45	258
	IsoformX2	13703	2035	1713	570	14	47	261
	IsoformX3	2188	2081	1937	645	12	45	13
	IsoformX4	19153	3275	3024	1007	24	145	107
	IsoformX5	27606	2587	2445	814	20	24	119
	IsoformX6	2280	1840	1565	521	13	167	109
	IsoformX7	9113	1697	1410	469	13	138	150
**Ovary**	IsoformX8	19791	2481	894	753	18	104	114
	IsoformX9	29945	1712	1518	505	12	86	108
	IsoformX10	136214	399	288	95	5	2	109
	IsoformX11	29646	1873	1762	505	12	245	110
**Testes**	IsoformX12	18316	2519	2268	755	19	135	116
**Gill**	No isoform found							

^#^ Sequence obtained from NCBIAceView database (https://www.ncbi.nlm.nih.gov/ieb/research/acembly/)

### Domain Identification in A2ML

**Fig 2:**
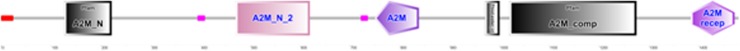
Functional domains of *Hilsa* A2ML protein (Consensus), determined by SMART tool. Six functional domains are shown with significant cutoff values i.e A2M_N (position 126–221; E value: 4e^-17^), A2M_N_2 (498–647; E: 9.23e^-39^), A2M (749–838; E: 1.19e^-41^), Thiol-ester_cl (999–1028; E: 4e^-15^), A2M_comp (1048–1303; E: 1.1e^-65^) and A2M_recep (1412–1500; E: 1.05e^-33^).

In comparison to *Homo sapiens* A2ML1 protein (Accession No: NP_000005.2), total six functional domains were identified in full length consensus transcript in present study (Table 2). These domains in A2ML consensus protein, predicted by SMART tool, are shown in Fig 2 with significant cutoff values i.e MG2 macroglobulin domain (A2M_N; Position 126–221), Alpha-2 macroglobulin family N-terminal region (A2M_N_2; 498–647), Alpha-2 macroglobulin family C-terminal region (A2M; 749–838), Thiol-ester_cl region (999–1028), Macroglobulin Complement component (A2M_comp; 1048–1303) and Macroglobulin receptor (A2M_recep; 1412–1500). Upstream of thioester motif, the Bait domain (a short stretch of about 40 amino acid residues) and beta-alpha signal sequence (Figure B in S1 File) were identified. At the superfamily level, A2ML consensus protein belonged to two super families- Terpenoid cyclases/ Protein prenyl transferases (Complement components family, 1.38e^-80^) and Alpha-macroglobulin receptor domain (A2M family, 3.14^e-33^). The longest isoform in liver (IsoformX1) showed all domains, while smallest isoform (IsoformX10) found in ovary contained only one domain (A2M_receptor). Multiple sequence alignment of *T*. *ilisha* consensus A2ML deduced protein with that of *Homo sapiens* (Accession no. NP_000005.2), *Danio rerio* (NP_001132951.1) *and Clupea harengus* (XP_012689768.1) revealed 45.1%, 53.2% and 72.8% similarities in A2M_Ndomains; 59.3%, 77.7% and 92.6% in A2M; 50.5%, 67.2% and 83% in A2M_2 and complement and 43.9% with *Homo sapiens* and 52% with *Danio rerio* in receptor domain, respectively (Figure C in S1 File).

**Table 2:** Domains identified in A2ML isoforms (*Tenualosa ilisha)* as compared to *Homo sapiens* (Accession No.XM_017018870.1).

Organism/Tissue	ID	No of domains	A2M_N (amino acid position)	A2M_N2 (amino acid position)	Bait region	A2M (amino acid position)	Thiol-ester cl (amino acid position)	A2M_Comp (amino acid position)	A2M_Recep (amino acid position)
***Homo sapiens***	A2ML1.a^***#***^	**6**	121–238	453–601	690–728	736–826	959–988	1008–1253	1352–1449
***T*. *ilisha***	**Consensus**	**6**	126–221	498–647	723–770	777–866	999–1028	1048–1303	1412–1500
**Liver**									
IsoformX1	20636	**3**	126–221	470–619	695–742	749–838	971–1000	1020–1275	1384–1472
IsoformX2	13703	**2**	126–221	471–554	-	-	-	-	-
IsoformX3	2188	**2**	126–221	498–647	-	-	-	-	-
IsoformX4	19153	**3**	-	6–155	230–266	273–362	495–524	544–799	910–998
IsoformX5	27606	**4**	-	-	-	100–189	322–351	371–602	713–801
IsoformX6	2280	**3**	-	-	-	-	1–23	43–298	409–497
IsoformX7	9113	**3**	-	-	-	-	1–23	43–274	385–470
**Ovary**									
IsoformX8	19791	**2**	-	-	1–21	22–111	244–273	310–547	658–682
IsoformX9	29945	**3**	-	-	-	-	1–23	43–298	409–497
IsoformX10	136214	**1**	-	-	-	-	-	-	1–87
IsoformX11	29646	**3**	-	-	-	-	75–104	124–379	490–578
**Testes**									
IsoformX12	18316	**4**	-	-	1–21	22–111	244–273	293–548	659–747

^**#**^ Sequence obtained from NCBIAceView database from https://www.ncbi.nlm.nih.gov/ieb/research/acembly/.

**A2M_N**: MG2 domain of alpha-2-macroglobulin

**A2M_N_2**: Alpha-2-macroglobulin family N-terminal region

**A2M**: Alpha-2-macroglobulin family

**Thiol-ester_cl**: Alpha-2-macroglobulin thiol-ester bond forming region

**A2M_Comp**: A-macroglobulin complement component

### Primary structure of A2ML consensus transcript

Prot-Param analysis of consensus A2ML sequence revealed a total of 1508 amino acids, 166.1 kD molecular weight and Iso-electric point (pI) of 5.98, which is indicative of protein’s acidic nature. A total of 149 negatively charged (Asp + Glu) and 128 positively charged residues (Arg + Lys) were identified. Aliphatic index and instability index (II) were computed to be 87.78 and 41.87, respectively. An extinction coefficient was 155075 M^-1^ Cm^-1^ on basis of cystine residues at 280 nm wavelength. The Grand average of hydro-pathicity (GRAVY) was -0.092. A total of 26 disulphide bonds was predicted by the web server DiANNA, which contains cysteinyl residues in A2ML protein (Table C in S1 File). NetPhos 3.1 server predicted141 phosphorylation sites at serine residues, 84 at threonine and 25 at tyrosine residues. Total eight potential N-glycosylation sites were identified at different positions containing unique Asn-Xaa-Ser/Thr sequences (Table D in S1 File). SignalP analysis predicted the single peptide sequence (1–22 aa) in A2ML, with cleavage site at 23 amino acid position. However, no nuclear localization signal was found in PSORT analysis, which indicates its localization to be cytoplasmic with 76.7% reliability.

### Characteristics of splice-junction

**Fig 3:**
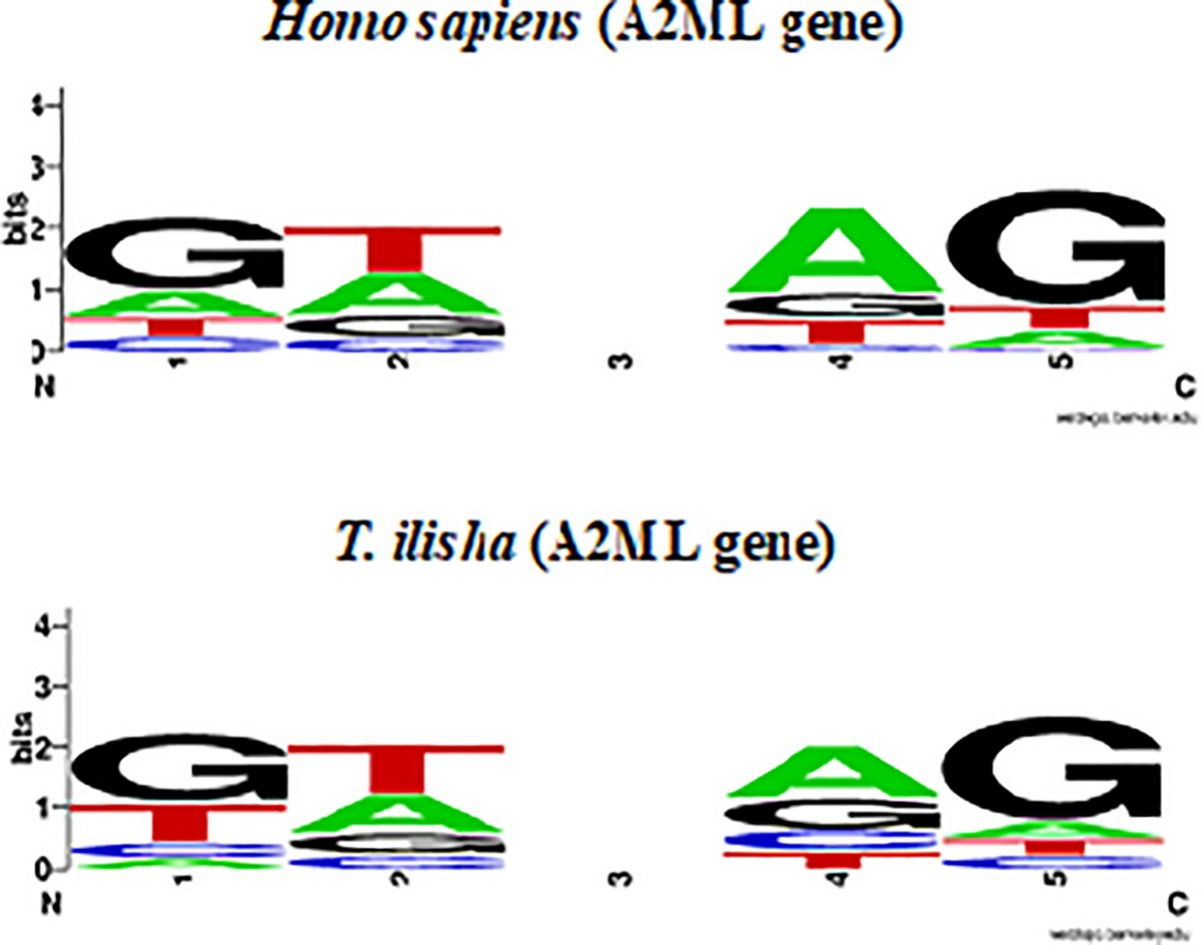
Frequency of nucleotides at splice junction in *T*. *ilisha* A2ML transcripts in comparison to *Homo sapiens* A2ML gene transcripts. Diagram depicts the similar pattern of nucleotides (GT-AG) at splice sites in both *Tenualosa ilisha and Homo sapiens*.

A total of 35 splice siteswere identified in *T*. *ilisha* A2ML consensus transcript (Fig 3 and Table E in S1 File) and nucleotides at nine out of 35 splice sites were common (AT-CC, TG-AG, TA-AG, GA-TG, GA-AG, GA-AG, GA-TG, GC-AG and CT-AG) in both *T*. *ilisha* and *Homo sapiens*. In *T*. *ilisha*, highest frequency (55.7%) of nucleotides at splice junction sites of A2ML gene was observed for GT-AG; while in lower range were AC-TC (10.7%) and CC-CC (7.9%).

### Putative markers for EUS

For finding the signatures for EUS disease response through comparison of A2ML proteins in resistant and susceptible species (Table 3; Figure D in S1 File), differential pattern of amino acids were observed at five positions 212, 269, 472, 567 and 906 (Table 3). The 218 amino acid position (I) was found in MG2 domain of alpha-2-macroglobulin (A2M_N domain) and at other two 494 and 594 positions (A and S) were in A2M_N_2 domain. In present study, one of the five markers (218:I) which is present in all 3 resistant species, was also observed in another group of species, which are not experimentally proven to be susceptible/resistant to *Aphanomyces sp* (Table 3). These fishes also showed synonymous and non-synonymous changes at two positions (970 and 594), when compared to EUS resistant fishes.

**Table 3:** Potential disease resistant markers for Epizootic ulcerative syndrome in fishes.

Fish species	Accession No	Family	Amino acid mutations at five positions ^#^					Resistant/ Susceptible	References ^$^
			I	II	III	IV	V		
			212	269	472	567	906		
***Cyprinus*** ***carpio***	BAA85038.1	Cyprinidae	I	I	A	S	Y	Resistant	[25, 27, 34,35]
***Oreochromis niloticus***	XP019212731.1	Cichlidae	I	I	A	S	Y	Resistant	[26]
***Maylanadia zebra***	XP014269643.1	Cichlidae	I	I	A	S	-	Putative Resistant	[36]
***Takifugu rubripes***	XP011616382	Tetraodontidae	*	A	Q	**.**	:	Unknown	[36]
***Poecilia reticulata***	XP008423358	Poeciliidae	*	Y	R	*	:	Unknown	[38]
***Labrus*** ***bergylta***	XP020512318	Labridae	*	*	R	**.**	:	Unknown	[36]
***Larimichthys crocea***	XP019131243	Sciaenidae	*	A	:	*	:	Unknown	[36]
***Monopterus albus***	XP020476381	Synbranchidae	*	A	K	**.**	:	Unknown	[36]
***Danio rerio***	NP001132951.1	Cyprinidae	S	P	:	**.**	N	Unknown	[36]
***Rachycentron canadum***	AIT68782.1	Rachycentridae	:	-	Q	**.**	:	Unknown	[36]
***Fundulus heteroclitus***	XP021166416	Fundulidae	*	S	R	**.**	:	Susceptible	[36,39]
*Clupea harengus*	XP012689768.1	Clupeidae	:	A	K	R	:	Susceptible	[36]
*Ctenopharyngodon idella*	AAR00337.1	Cyprinidae	-	:	:	**.**	:	Susceptible	[40]
*Salmo salar*	XP014069237.1	Salmonidae	T	A	K	**.**	:	Susceptible	[41]
*Carassius gibelio*	AGU16534.1	Cyprinidae	Y	**.**	**.**	M	E	Susceptible	[26,36]
*Esox lucius*	XP010891963	Esocidae	R	-	E	R	:	Susceptible	[42]
***Tenualosa ilisha***	Present study	Clupeidae	R	:	K	**.**	:	Unknown	Study species

^**#**^Position of five putative markers (amino acids) in A2ML1 protein of *Danio rerio*: **I– 212 (I→S); II– 269 (I→P); III– 472 (A→T); IV– 567 (S→G); V– 906 (Y→N)**

^**$**^ References are given in Manuscript

'*' indicates fully conserved residue.

**':'** indicates synonymous residues.

**'.'** indicates non-synonymous residues.

**Fig 4:**
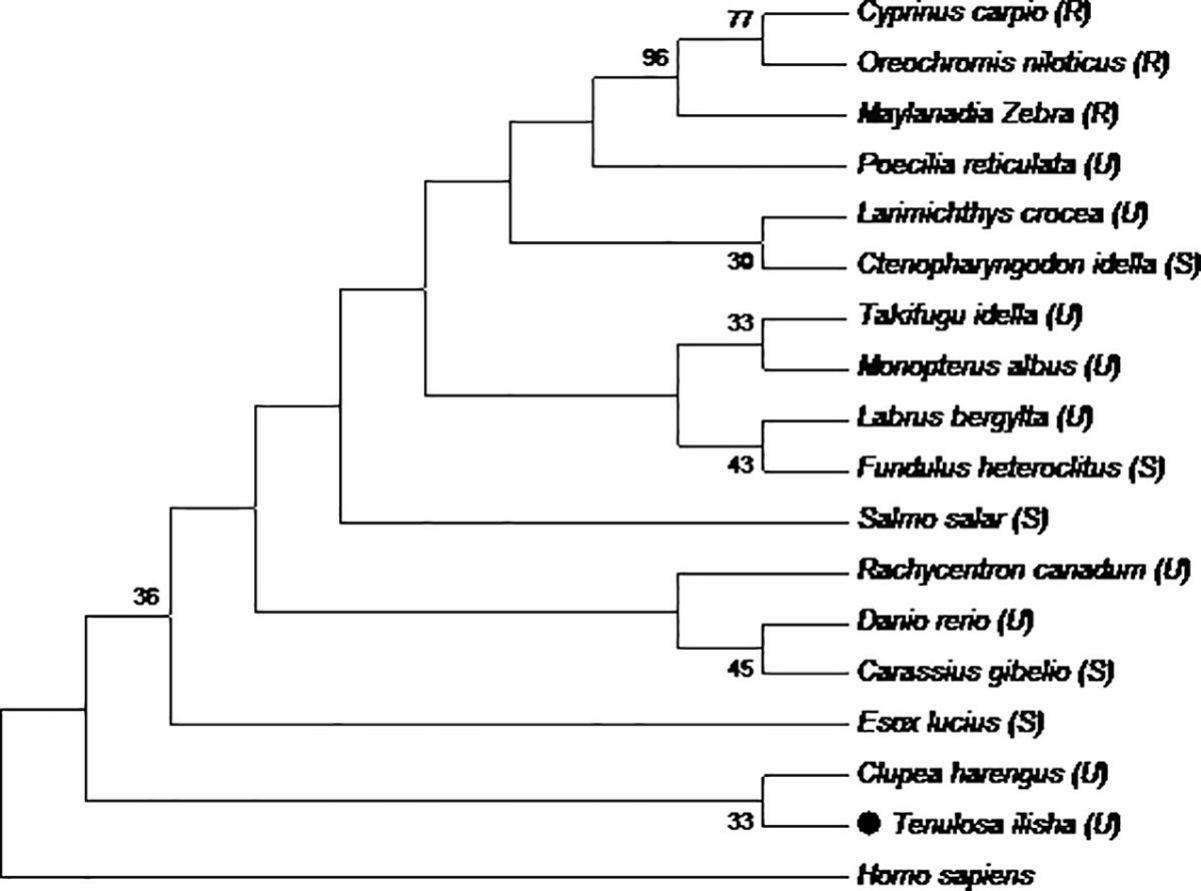
Phylogenetic analysis based on EUS markers in A2ML transcript from disease resistant (R), susceptible (S) and unknown (U) fish species including study species *T*. *ilisha* (shown in black circle). *Homo sapiens* is taken as the outgroup and node support is indicated by bootstrap values. The scale bar represents 0.05 substitutions per site.

Grouping through phylogenetic analysis based on these markers classified fishes into two major groups (Fig 4), one group includes EUS resistant fishes, i.e. *Cyprinus carpio*, *Oreochromis niloticus* and *Maylanadia zebra*, separated from other group of susceptible and unknown response, with significant bootstrap values (96%). Both susceptible fish with unknown response against EUS disease distributed in second group (Fig 4).

## Discussion

Alpha-2- macroglobulin (A2M) is an important component of innate immune system, which has a protective role in both vertebrate and invertebrates against invading pathogens [3]. The present study, for the first time, reported the twelve isoforms of A2M in four tissues of *T*. *ilisha*. The liver is an impotent immune tissue and has balance between immunity and tolerance due to dynamic interactions between the number of immune cells in the liver [43], while fish gill is a mucosa-associated lymphoid tissue, in which local immune responses are provided by leukocyte populations [44]. The immune abilities are crucial in fish ovary for facilitation of constant removal of degenerating germ cells [45], whereas in testis for prevention autoimmune diseases and chronic inflammation [46]. The long read sequencing technique sequence entire cDNA (transcripts) without PCR amplification or fragmentation/assembly and provides accurate information about full complement of isoforms up to 10 kb size [47,48]. The short-read sequencing methods are not optimal for identifying alternatively transcribed transcripts and unable to distinguish between highly similar gene isoforms [49]. Earlier studies have used traditional methods of cloning and Sanger sequencing using rapid amplification of cDNA ends (RACE) techniques in identification of A2M isoforms in *Cyprinus carpio* [6,14], Giant fresh water prawn, *Macrobrachium rosenbergii* [2], shrimp *Fenneropenaeus chinesis* [15], white shrimp *Litopenaeus vannamai* [50]. Identification of novel and full length isoforms using PacBio sequencing approach have been reported in animal, plants and microbes [48,49].

Alignment of amino acid sequences revealed the presence of six functional domains in *T*. *ilisha*A2ML protein as also found in that of human and the bait region, which is a cleavage site of unique amino acid sequence and susceptible to specific proteases, showed very little similarity in both[51]. The A2M activity is depended on proteolytic cleavage of Bait region by host/pathogen's proteases, which inhibit protease [3]. Divergence in the Bait region has been reported in vertebrates and invertebrates [3,14,15,52]. High diversity in bait region provides A2M, the ability to recognize wide range of proteases and expands the diversity of immune recognition and protease inhibition [15]. However, in the present study, Bait region was observed only in few isoforms of tissues studied, pointing out that not all the isoforms detected in the present study may be functionally active for immune function. Similar situation was observed in FLICE isoforms, where only two out of eight isoforms were functionally active [53]. However, some isoforms lacking bait region showed the presence of receptor domain, which helps in binding to macrophase and fibroblasts during phagocytosis process [54,15] which indicates that they are not functionally redundant (inhibitory function), but may have role in other biological processes. Thus, the presence of bait region in only four out of twelve isoforms found in present study calls for further studies, required to validate the function of these isoforms.

In the present study, different splice variants were found in different tissues and thus splicing of A2M mRNA seemed tissue-specific. Similar patterns of differential distribution across various cell types in mice was also observed by Peng et al [55], which indicated that cell types core biological functions of the genes was correlated with the unique splicing pattern of specific cell type. There are reports of role of specific A2M isoforms in specific tissues against pathogens in challenging experimental studies. The multiple a2M isoforms (a2M1-4) with up-regulated expression of a2M3 isoform in liver was reported in European common carp, when challenged with the pathogen, *T*. *borreli* [14]. Studies in giant freshwater prawn (*Macrobrachium rosenbergii) by* Likittrakulwong et al [2] indicated high expression level of *Mr-*2α2M isoform than *Mr-*1α2M in hemocytes and hepatopancreas after infection with *Aeromonas hydrophila*, which represented the specific role of this isoform in protection. Similarly, high expression of FcA2M-1 isoform (out of two other isoforms, FcA2M-2 and FcA2M-3) was reported in hemocytes and lymphoid organ of Chinese shrimp (*F*. *chinesis*) upon challenge to white spot syndrome virus (WSSV) and Vibrio pathogen [15]. Although alternative splicing in A2ML is observed at the transcript level in *T*. *ilisha*, further experiments are needed for the regulation of their expression and immune function.

It was interesting to find A2M isoforms transcripts in reproductive tissues, as it has been reported to be mainly produced by the liver. A novel reproductive tissue-associated A2M cDNA is known in humans and rats [56], however, it has not been reported in fish before this study. In cancerous ovary epithelial cells of the chicken studies by Lim et al [57] indicated *A2M* to be aestrogen-regulated and involved in switching to a cancerous state.

Disease resistance is one of the targets forselective breeding programmes. However, unavailability of suitable phenotypes as well as the candidate gene selection can hamper these programmes [58]. Only few fish species are known to be resistant to EUS, these species can form the necessary basic material for identification of genetic variation, contributing towards disease resistance. In the present study, the five markers identifiedin different fish species can form a signature for response to EUS disease. Potential markers predicted *T*. *ilisha* to be in EUS susceptible category. Interestingly, on the basis of these markers, *Maylandiazebra* (family Cichlidae), an ornamentalfish of unknown response to EUS, grouped with resistant species *Cyprinus carpio* (Cyprinidae family) and *Oreochromis niloticus (*Cichlidae family). Another member of same family, Mayan cichlid, *Cichlasoma urophthalmus* has also been reported to be EUS resistant in challenging experiment with *A*. *invadans* infection [59]. Moreover, other fishes used in present study grouped differently from resistant fishes in marker based grouping, which may point out to the possibility of these fishes to be susceptible to moderately susceptible to *A*. *invadans* infection, however, challenging experiments are needed to be conducted to verify the conclusion for each species. On the basis of functions of A2M of protecting against infections, Rehman et al [60] has also suggested it be potential biomarker for the prognosis and diagnosis of diseases. Earlier studies have also identified signature markers associated with different production traits, like growth performance [61,62,63] and meat quality [64].

## Conclusion

The present findings revealed a vast diversity in tissue specific A2M transcripts in *T*. *ilish*a, which may serve as a vital genomic resource, to uncover new biological functions of alternate splicing and to generate important insights into mechanisms of fish immune response to various diseases. Putative markers identified in A2M for differential response to EUS in this study may help in developing the new tools in detection of loci (genomic region) involved in genetic variation for disease resistance. To confirm the role of these A2M isoforms in response to EUS disease susceptibility/resistance, further experimental studies are needed.

## Supporting information

S1 File Supporting tables and figures. (DOC)
