# Supplementary material for: Tissue specific alpha-2-Macroglobulin (A2M) splice isoform diversity in Hilsa shad, Tenualosa ilisha (Hamilton, 1822)
Source: PLoS One. 2019 Jul 23;14(7):e0216144. doi: 10.1371/journal.pone.0216144 (PMC6650032; doi:10.1371/journal.pone.0216144)
Supplement: S1 File — (DOC) [file pone.0216144.s001.doc]

**Table A. PacBio transcriptome data generated from four tissues of *Tenualosa ilisha* (Liver, Gill, Ovary and Testes)**

| **Tissues** | **Liver** | **Gill** | **Ovary** | **Testes** |
| --- | --- | --- | --- | --- |
| Data generated | 5.99 Gb | 4.82 Gb | 4.21 Gb | 5.80 Gb |
| Full length reads | 28,351 | 38,232 | 17,429 | 30,696 |
| BioSample | SAMN07977427 | SAMN07977429 | SAMN07977431 | SAMN07977430 |
| SRA accession | SRR6277490  to  SRR6277500 | SRR6418035  to  SRR6418045 | SRR6433061  to  SRR6433071 | SRR6432945  to  SRR6432955 |

**Table B. Sequence similarity of *Tenualosa ilisha* A2ML gene with other fishes in Blastx search at NCBI database**

| **Name of Species** | **Accession Number** | **Description** | **Identity** | **Query coverage** | **E value** |
| --- | --- | --- | --- | --- | --- |
| *Clupea harengus* | XP_012689768.1 | Predicted: alpha-2-macroglobulin-like protein 1 isoform X1 | 72% | 90% | 0.0 |
| *Cyprinus carpio* | BAA85038.1 | alpha-2-macroglobulin-1 | 56% | 95% | 0.0 |
| *Danio rerio* | NP_001132951.1 | alpha-2-macroglobulin-like precursor | 55% | 95% | 0.0 |
| *Ctenopharyngodon idella* | AAQ74974.1 | Predicted: alpha-2-macroglobulin | 55% | 84% | 0.0 |
| *Homo sapiens* | NP_000005.2 | alpha-2-macroglobulin isoform a precursor | 38% | 95% | 0.0 |

**Table C.** Disulphide bond prediction in A2ML protein by DIANNA 1.1 tool

| **S.No** | **Cysteine Seq position** |  | **Bond** |
| --- | --- | --- | --- |
|  | 15 - 330 | 315 | GGFLSCLFTLC-MDGSGCAPHSF |
|  | 15 - 635 | 620 | GGFLSCLFTLC-QPGSLCALSAV |
|  | 15 - 859 | 844 | GGFLSCLFTLC-NYLPNCIMVTV |
|  | 15 - 1357 | 1342 | GGFLSCLFTLC-VKGSACASMGV |
|  | 251 - 330 | 79 | VQVQVCAKYDM-MDGSGCAPHSF |
|  | 251 - 635 | 384 | VQVQVCAKYDM-QPGSLCALSAV |
|  | 251 - 1357 | 1106 | VQVQVCAKYDM-VKGSACASMGV |
|  | 278 - 330 | 52 | CFFNSCVARYT-MDGSGCAPHSF |
|  | 278 - 635 | 357 | CFFNSCVARYT-QPGSLCALSAV |
|  | 278 - 1357 | 1079 | CFFNSCVARYT-VKGSACASMGV |
|  | 297 - 723 | 426 | ADLSLCRDVKP-LQIPDCLTFLG |
|  | 318 - 330 | 12 | EPQAPCITVSV-MDGSGCAPHSF |
|  | 318 - 635 | 317 | EPQAPCITVSV-QPGSLCALSAV |
|  | 318 - 810 | 492 | EPQAPCITVSV-ETEAFCLSPQG |
|  | 584 - 1357 | 773 | QVLVYCILPSE-VKGSACASMGV |
|  | 603 - 635 | 32 | FSTEKCFKNKV-QPGSLCALSAV |
|  | 603 - 679 | 76 | FSTEKCFKNKV-EDHVECFKVRP |
|  | 603 - 723 | 120 | FSTEKCFKNKV-LQIPDCLTFLG |
|  | 603 - 859 | 256 | FSTEKCFKNKV-NYLPNCIMVTV |
|  | 603 - 921 | 318 | FSTEKCFKNKV-HSEALCGNEVV |
|  | 603 - 1357 | 754 | FSTEKCFKNKV-VKGSACASMGV |
|  | 635 - 921 | 286 | QPGSLCALSAV-HSEALCGNEVV |
|  | 635 - 1010 | 375 | QPGSLCALSAV-KMPYGCGEQNM |
|  | 635 - 1504 | 869 | QPGSLCALSAV-EYSFPCAGDRX |
|  | 887 - 1357 | 470 | YSSCLCAHERK-VKGSACASMGV |
|  | 959 - 1357 | 398 | FNWLLCPKGGA-VKGSACASMGV |

**Table D.** Predicted potential N-glycosylation sites in deduced protein of Hilsa A2ML

| **S.No** | **Sequence** | **Positions** |
| --- | --- | --- |
| **1** | NETL | 54 |
| **2** | NYSL | 377 |
| **3** | NDTA | 408 |
| **4** | NMTV | 414 |
| **5** | NLTT | 432 |
| **6** | NVTV | 907 |
| **7** | NMTV | 1147 |
| **8** | NITV | 1400 |

**Table E.** Characteristics of splice junction in Hilsa A2ML sequence (Consensus) as compared to Human A2ML transcript

| **Exons** | **Human** | **Hilsa** | **Exons** | **Human** | **Hilsa** |
| --- | --- | --- | --- | --- | --- |
| **E1** | AT-CC | AT-CC | **E19** | AA-GG | GT-GG |
| **E2** | AA-TT | CT-TT | **E20** | GT-GG | TG-GG |
| **E3** | GT-AG | CA-AG | **E21** | GT-AA | GA-AG |
| **E4** | TG-AG | TG-AG | **E22** | CC-AG | GC-AG |
| **E5** | TA-AG | TA-AG | **E23** | GA-GG | GG-GG |
| **E6** | GA-TG | GA-TG | **E24** | GA-AG | GA-CG |
| **E7** | TG-TA | TT-CA | **E25** | GG-TG | GA-TG |
| **E8** | GG-AG | GG-GG | **E26** | GC-AG | GC-AG |
| **E9** | AC-AG | AT-AG | **E27** | GG-AT | GG-AA |
| **E10** | GT-AG | GT-CA | **E28** | GA-AG | GT-AC |
| **E11** | AT-AG | TT-AG | **E29** | GA-AG | TT-AG |
| **E12** | GG-AT | TA-TT | **E30** | GA-AG | GA-GG |
| **E13** | TT-AG | AT-GT | **E31** | AC-AG | GT-GG |
| **E14** | GA-AG | GA-AG | **E32** | TT-TA | TA-CT |
| **E15** | GT-CT | GT-AG | **E33** | CT-AG | CC-CG |
| **E16** | GT-GG | GT-AA | **E34** | CT-AG | CT-AG |
| **E17** | GA-TG | GA-TG | **E35** | AT-GA | TG-GC |
| **E18** | CA-GT | TC-GG |

| 1 1 63 6 123 26 183 46 243 66 303 86 363 106 423 126 483 146 543 166 603 186 663 206 723 226 783 246 843 266 903 286 963 306 1023 326 1083 346 1143 366 1203 386 1263 406 1323 426 1383 446 1443 466 1503 486 1563 506 1623 526 1683 546 1743 566 1803 586 1863 606 1923 626 1983 646 2043 666 2103 686 2163 706 2223 726 2283 746 2343 766 2403 786 2463 806 2523 826 2583 846 2643 866 2703 886 2763 906  I  II  III  IV | acacagaagtgtgtgtgtgtgtgcagacagagagtctcaacagacta***ATG***GCCCTCTCCAGG      M  A  L  S  R  ATCCTGATTGCTGGGGGGTTCCTGAGCTGCCTCTTCACCCTCTGCTTGGCCACCAAAACA  I  L  I  A  G  G  F  L  S  C  L  F  T  L  C  L  A  T  K  T  CCAGAGCCGTCTTTCCTGGTGACCTTTCCTGCTCTAATAAGTTCAGAGTCGAAAGCCAAA  P  E  P  S  F  L  V  T  F  P  A  L  I  S  S  E  S  K  A  K  CTGTGTGCCAGCCTCCTGAGTCCAAATGAGACTCTGGTCATGTCCATCTATCTGACCCAT  L  C  A  S  L  L  S  P  N  E  T  L  V  M  S  I  Y  L  T  H  GGAGACCAGAGGAAGATGCTGCACCAGGAAACGTCAGACAAAGACTTTCACAGCTGCTTC  G  D  Q  R  K  M  L  H  Q  E  T  S  D  K  D  F  H  S  C  F  CACTTCCAGGCCCCTTCGGTTAATATGGGCTCTGTGCAGAAAATCCAAGTAGAAGTTAAA  H  F  Q  A  P  S  V  N  M  G  S  V  Q  K  I  Q  V  E  V  K  GGTCAAACATCTAACTGGGCTTTTGACAGTCGGGTGAAATTCAGACCAAAACCTTCCCCC  G  Q  T  S  N  W  A  F  D  S  R  V  K  F  R  P  K  P  S  P  ATAACCTTCATCCAGACAGACAAGCCCATATACAGCCCTGGCCAAACAGTGCACTTCAGA  I  T  F  I  Q  T  D  K  P  I  Y  S  P  G  Q  T  V  H  F  R  GTGGTCACCATGGACCCAGACTTCATTCCCCTCAGTGAAGAGTACCCCCACATGTCTCTG  V  V  T  M  D  P  D  F  I  P  L  S  E  E  Y  P  H  M  S  L  CAGGACAGCCGGGGCAACGTTATTGGCCAGTGGCTCAATGTGAGATCACAGGGCAAGATA  Q  D  S  R  G  N  V  I  G  Q  W  L  N  V  R  S  Q  G  K  I  GTGCAGCTGTCTCATGTGTTAAACCCAGAGGCCCCTCAGGGCAAATATACACTCAAAATC  V  Q  L  S  H  V  L  N  P  E  A  P  Q  G  K  Y  T  L  K  I  AATGGCAAACAAACATCAATTCAGACTCGGACCCATGAGTTTGAAGTGAAAAAATATGTG  N  G  K  Q  T  S  I  Q  T  R  T  H  E  F  E  V  K  K  Y  V  TTGCCCAAGTTTGATGTTACACTAACGACTCCAAAGGACATCAGTGTTGGAGCGGAAGCA  L  P  K  F  D  V  T  L  T  T  P  K  D  I  S  V  G  A  E  A  GTCCAAGTGCAAGTTTGTGCAAAGTATGACATGGCTACAGAAGCATGGAAGGTGGTGGTC  V  Q  V  Q  V  C  A  K  Y  D  M  A  T  E  A  W  K  V  V  V  GATCAGAGCCATTTTCTCCTTTGTTTTTTTAACTCTTGTGTTGCCAGGTACACATTTGGT  D  Q  S  H  F  L  L  C  F  F  N  S  C  V  A  R  Y  T  F  G  CAGCCAGTGCCAGGGAAAGCTGACCTGTCGCTGTGTCGAGATGTTAAACCTTATCGAGAT  Q  P  V  P  G  K  A  D  L  S  L  C  R  D  V  K  P  Y  R  D  TACGAAGACGAATCCACTGCAGAGCCACAGGCACCTTGTATAACCGTGTCAGTATGGATG  Y  E  D  E  S  T  A  E  P  Q  A  P  C  I  T  V  S  V  W  M  GATGGGTCTGGTTGTGCGCCTCATTCATTTAGCATGGCCATGTTTACACAGCCTGAGTTC  D  G  S  G  C  A  P  H  S  F  S  M  A  M  F  T  Q  P  E  F  AATAAAAAACTCAAGAACATGTTAAGATTCAGTGCCACAGTAACTGAGGAAGGAACAGGT  N  K  K  L  K  N  M  L  R  F  S  A  T  V  T  E  E  G  T  G  ATCAGTCGGGCCGACAGCAAAGACATTGATTTGAACTACTCTCTTGGGAAACTTACATTT  I  S  R  A  D  S  K  D  I  D  L  N  Y  S  L  G  K  L  T  F  ATTGATACACTCGACGTGATTGAGGATGGAGCAGTTTTGGAAGGCAAGATCAAAGCGACC  I  D  T  L  D  V  I  E  D  G  A  V  L  E  G  K  I  K  A  T  CATTACAACGACACGGCCATCGCTAACATGACGGTCCACCTGTTTAATGGAGTGGGTCTG  H  Y  N  D  T  A  I  A  N  M  T  V  H  L  F  N  G  V  G  L  GGTGCAACGCTCCTGCTGAACCTGACCACAGACTCTGATGGAGTGGCCCAGTTCTCTCTC  G  A  T  L  L  L  N  L  T  T  D  S  D  G  V  A  Q  F  S  L  AACACGGCTCCTTTCCTTGGAGACTTTAAGCTCACTGCAAGTACATCAACCGATATTGAT  N  T  A  P  F  L  G  D  F  K  L  T  A  S  T  S  T  D  I  D  GAATATATTCCATATGAAACTCCACATTATGACTCTGCAACAAAGTCTGTGTCCAGGCTA  E  Y  I  P  Y  E  T  P  H  Y  D  S  A  T  K  S  V  S  R  L  AGACCAGCGCTTGTACACATACCATCATCCAGCTCTCTGACCATAAAGCCTTTAGATGAT  R  P  A  L  V  H  I  P  S  S  S  S  L  T  I  K  P  L  D  D  GACCTTCCATGTGGTAAAGACGTAGCCATCACTACTAAGTACGTTTTCATTGGAGAGACG  D  L  P  C  G  K  D  V  A  I  T  T  K  Y  V  F  I  G  E  T  TTCAACACAGACCATGTGGATATCGTATACATGGCTTTATCCAAAGGAGAGATTGTTCAT  F  N  T  D  H  V  D  I  V  Y  M  A  L  S  K  G  E  I  V  H  CATGGCTTCACTGAGCCACCAGTGCAGGGTTCCCAACCAGTGACTGAGGGTGAGGTGTCC  H  G  F  T  E  P  P  V  Q  G  S  Q  P  V  T  E  G  E  V  S  TTCCAGGTGCCCATCAGAGCTGAGATGGCCCCCTCAGTGCAGGTCCTGGTCTACTGTATC  F  Q  V  P  I  R  A  E  M  A  P  S  V  Q  V  L  V  Y  C  I  TTACCCAGTGAGACGGTCCTGGCTGATAGCAGAACCTTCTCAACAGAGAAGTGCTTTAAA  L  P  S  E  T  V  L  A  D  S  R  T  F  S  T  E  K  C  F  K  AATAAGGTGGCGCTGCAGTTCTCTCCCCCTAAAGCTGTTCCTGGAGAGCAGAACTCCCTG  N  K  V  A  L  Q  F  S  P  P  K  A  V  P  G  E  Q  N  S  L  CAGATATCAGCTCAGCCTGGCTCTCTGTGTGCCCTCAGTGCTGTGGACCAGAGCATCTTC  Q  I  S  A  Q  P  G  S  L  C  A  L  S  A  V  D  Q  S  I  F  ATCCTGGAACCAGGGAAACGCCTGGATGTACAAAAGGTCTTTGGATTATTACCAGAGTTG  I  L  E  P  G  K  R  L  D  V  Q  K  V  F  G  L  L  P  E  L  AACTCAGAAGTTCATTATGATGCTGAAGATCATGTAGAGTGCTTTAAAGTCAGGCCGAGG  N  S  E  V  H  Y  D  A  E  D  H  V  E  C  F  K  V  ***R* P* R****  CGATCCTCAATGCCATACCATTATCCACGGCACTCTGATAGTGGGGTCTCTAAAGTTTTC ***R**** S  S  M  P  Y  H  Y  P  R  H  S  D  S  G  V  S  K  V  F  AAGATATTGGGACTGAAGACTATTTCAAATTTGGCACTGCAGATCCCTGACTGCCTAACA  K  I  L  G  L  K  T  I  S  N  L  A  L  Q  I  P  D  C  L  T  TTCTTGGGACGTAGATATTACTTGAATCATGGATATCGTGTTATTCAGCATGAGCATGGT  F  L  G  R  R  Y  Y  L  N  H  G  Y  R  V  I  Q  H  E  H  G  TATATGGTCTTGTCTTCTTCTGGAGTGGGAGGAGGGATGTTTGGACCTTCAAGAGAGGAG  Y  M  V  L  S  S  S  G  V  G  G  G  M  F  G  P  S  R  E  E  TTTGGCGTGACTGTTCGTACAGTCTTCCCAGAGACTTGGATCTGGGACCTGGCAGAAGTT  F  G  V  T  V  R  T  V  F  P  E  T  W  I  W  D  L  A  E  V  GGGGTGTCTGGAGAAGCGCAACTCCCTCTGACTGTCCCTGACACCATCACCACCTGGGAG  G  V  S  G  E  A  Q  L  P  L  T  V  P  D  T  I  T  T  W  E  ACGGAAGCTTTCTGTCTGTCCCCCCAGGGCTTTGGTCTGGCCCCACCTGTTCAGCTCACA  T  E  A  F  C  L  S  P  Q  G  F  G  L  A  P  P  V  Q  L  T  GTTTTCCAGCCATTCTTCCTGGAGCTCTCCCTGCCATACTCCATCATCCGTGGAGAGACC  V  F  Q  P  F  F  L  E  L  S  L  P  Y  S  I  I  R  G  E  T  TTTCAGCTAAAAGCAACAGTCTTCAACTACCTACCCAACTGTATCATGGTGACAGTGACT  F  Q  L  K  A  T  V  F  N  Y  L  P  N  C  I  M  V  T  V  T  CCAGCTCCTTCATCAGACTTCACTTTGACCCCTTCCTATGATGGCCAGTACTCCTCCTGT  P  A  P  S  S  D  F  T  L  T  P  S  Y  D  G  Q  Y  S  S  C  CTGTGTGCACATGAGAGGAAGACCTTCACATGGACACTGGTGCCCTCTGTGCTGGGACTG  L  C  A  H  E  R  K  T  F  T  W  T  L  V  P  S  V  L  G  L  ATGAACGTTACAGTGAGTGCAAAGGCCAGTCACTCTGAGGCCCTGTGTGGCAATGAGGTT  M  N  V  T  V  S  A  K  A  S  H  S  E  A  L  C  G  N  E  V  ***Start*** | 2823 926 2883 946  2943 966 3003 986  3063 1006 3123 1026 3183 1046 3243 1066 3303 1086 3363 1106 3423 1126 3483 1146 3543 1166 3603 1186 3663 1206 3723 1226 3783 1246 3843 1266 3903 1386 3963 1306 4023 1326 4083 1346 4143 1366 4203 1386 4263 1406 4323 1426 4383 1446 4443 1466 4503 1486 4563 1506 4623 | GTGTCTGTTCCTGAGAGGGGACGCATAGACATAGTCACACGCCCCCTACTGGTAAAGGCC  V  S  V  P  E  R  G  R  I  D  I  V  T  R  P  L  L  V  K  A  GAAGGAACTGAGAAGACTGAAAGTTTCAATTGGCTTTTATGTCCAAAAGGGGGCGCTCTG  E  G  T  E  K  T  E  S  F  N  W  L  L  C  P  K  G  G  A  L  ACAGAGGAGGTGGAGCTGAAACTCCCACAGAATGTGGTGCAGGGATCAGCCAGAGCCTCA  T  E  E  V  E  L  K  L  P  Q  N  V  V  Q  G  S  A  R  A  S  GTGTCTGTGCTGGGAGACATCCTGGGTCGCGCACTGAAGAACATCGACAGCCTCCTGAAG  V  S  V  L  G  D  I  L  G  R  A  L  K  N  I  D  S  L  L  K  ATGCCTTATGGTTGTGGGGAGCAAAATATGGCCATCCTGTCCCCAAACATCTACATTCTG  M  P  Y  G  C  G  E  Q  N  M  A  I  L  S  P  N  I  Y  I  L  CAGTACCTACAGAACACTGGACAGCTCACAGCAGAAATTAGGGAAAAAGCAACAAATTTT  Q  Y  L  Q  N  T  G  Q  L  T  A  E  I  R  E  K  A  T  N  F  CTTAAAAACGGATATCAGAGACAGCTGAACTACAAGCACTTTAATGGAGCCTACAGCACA  L  K  N  G  Y  Q  R  Q  L  N  Y  K  H  F  N  G  A  Y  S  T  TTTGGCAGCGGAGAGGAAAACACATGGCTGACTGCATTTGTATTAAGGTCCTTTGGTAAA  F  G  S  G  E  E  N  T  W  L  T  A  F  V  L  R  S  F  G  K  GCAAAGTCCTTTGTCTACATTGACCCAGTCATAATTGAATCTGCAAAGACCTGGCTTCTA  A  K  S  F  V  Y  I  D  P  V  I  I  E  S  A  K  T  W  L  L  ACAAAGCTTCAACCAGATGGTGCTTTCTTCATGCAGGGAAAACTCTTTAACAACAGGATG  T  K  L  Q  P  D  G  A  F  F  M  Q  G  K  L  F  N  N  R  M  AAGGGAGGTGTAAGCGATGATTTGACAATAAGTGCCTACATTGCTGCAACACTGCTGGAG  K  G  G  V  S  D  D  L  T  I  S  A  Y  I  A  A  *T  L  L  E*  CTTAACATGACTGTGCCGGAACACACAATGGCGTTCCTGAAGTCCGGCTATAGTGGGTCA  *L  N  M  T  V  P  E  H  T  M  A  F  L  K  S  G  Y  S  G  S*  CCACCTCCTCCCAGAGACCTCTCCCATGCGAGAGAAATCCTGATGTTTCTAAGGCCGTCC *P  P  P  P  R  D  L  S  H  A  R  E  I  L  M  F  L  R  P  S*  ACTAATGATTTCTCCAACACCTACACCACTGCACTACTGGCCTACACCTTCAGTCTCGCT  *T  N  D  F  S  N  T  Y  T  T  A  L  L  A  Y  T  F  S  L*  A  GGAGAAGAGGACATCCGAGCTCAGCTTCTCAAACACTTGGACAGTGTAGCGACCTCTAGT  *G  E  E  D  I  R  A  Q  L  L  K  H  L  D  S  V  A  T  S  S*  GGGAATCTCCTGCACTGGTCTCAGTCCTCTTCAGAGCGAGCTGATTCCCTGGCGGTGGAG *G  N  L  L  H  W  S  Q  S  S  S  E  R  A  D  S  L  A  V  E*  ACCAGCTCTTATGTGCTGTTAGCTGTTCTCACGAAATCCACACTGACTGCTGCTGACTTG  *T  S  S  Y  V  L  L  A  V  L  T  K  S  T  L  T  A  A  D  L*  GGCTATGCTGCCAGGATTGTCAGCTGGCTGGTGAAGCAGCAGAATCCATATGGAGGCTTC  *G  Y  A  A  R  I  V  S  W  L  V  K  Q  Q  N  P  Y  G  G  F*  TCTTCCACACAGGACACAGTGGTGGCACTGCAGGCTCTGGCTCTCTACGCCACCAAGATC *S  S  T  Q  D  T  V  V  A  L  Q  A  L  A  L  Y  A  T  K  I*  TTCAACCCTCAAGGCTCCAGCACAGTGACAGTGCAGTCAGCAGGTGGCGCCAAACACCAG  F  N  P  Q  G  S  S  T  V  T  V  Q  S  A  G  G  A  K  H  Q  TTTGATGTGAACCAGCACAACACATTACTGTACCAGGAGAGGGCGCTGCTGGACGTTCCT  F  D  V  N  Q  H  N  T  L  L  Y  Q  E  R  A  L  L  D  V  P  GGGAAGTACAGCATCGAAGTGAAGGGCTCTGCATGTGCTTCTATGGGGGTGGCTCTTTTC  G  K  Y  S  I  E  V  K  G  S  A  C  A  S  M  G  V  A  L  F  TACAACATCCCCACTCCTACTGAGCACTCAACCTTGAGCATCAATACTAAGACCATTTGT  Y  N  I  P  T  P  T  E  H  S  T  L  S  I  N  T  K  T  I  C  CCGACTCCTCTGAACAAGGCCAATGGACAGGCCATCTACTTAAACATCACAGTCCGGTAT  P  T  P  L  N  K  A  N  G  Q  A  I  Y  L  N  I  T  V  R  Y  GATGGGCCTCTGCAGAGCACCAACATGGCCATCATTGATGTAACGATGCTGTCTGGCTTC  D  G  P  L  Q  S  T  N  M  A  I  I  D  V  T  M  L  S  G  F  ACTCCTGACTCTGGCACTGTTGAAAGACTCCAGAGCAGCAAATATGTGGACAGAGTCGAC  T  P  D  S  G  T  V  E  R  L  Q  S  S  K  Y  V  D  R  V  D  AAGAAAGATGACCACATTCTCGTGTATCTGTCAGCGCTTCCTAAGGCCATGTCCATTCAC  K  K  D  D  H  I  L  V  Y  L  S  A  L  P  K  A  M  S  I  H  TACCTCCTGATCATCCAACAGGATCTCCTAGTGAACAACCTGAAGCCAGCAGTGGTCAAG  Y  L  L  I  I  Q  Q  D  L  L  V  N  N  L  K  P  A  V  V  K  GTGTACGACTACTACCAGACCAGTGACCAAGCTGAGGCAGAGTACAGCTTTCCTTGTGCA  V  Y  D  Y  Y  Q  T  S  D  Q  A  E  A  E  Y  S  F  P  C  A  GGAGACCGC***tga***ggagagaggtcttcagtgacattcaacaaggacttcacgattagagat  G  D  R   1508 tttaaaatgaggtgaatgagtgaatacactgaaacatactaataaagaatgcttttac 4680  V  VI  VII  ***Stop*** |
| --- | --- | --- | --- |

**Figure A.** Nucleotide (4680 nt) and deduced amino acid (1508 a.a.) sequence of A2ML1 in *T. ilisha* showing seven domains (I-VII) containing sequences (shaded area). Domains A2M_N (I), A2M_N2 (II), Bait region (III), A2M (IV), A2M_2 (V), A2M_complement (VI) and A2M_receptor (VII) are represented in shaded background. Thiol-ester cl motif (GCGEQ) and beta–alpha processing signal (RPRR) are indicated by rectangular box and asterisks, respectively

**A2M_N domain**

Homo sapiens_NP_000005.2 129 VFVQTDKSIYK PGQTVKFRVV SMDENFHPLN ELIPLVYIQD PKGNRIAQWQ SFQLEGGLKQ FSFPLSSEPF QGSYKVVVQK KSGG--RTEH PFTV 221

Danio rerio_NP01132951 127 TFIQTDKPIYM PGQIVNFRVV TMDTNFAPVD QQYSIIVLED SQDNRIGQWT NVSSTRWILQ RSYELNPECR EGAYKLKAFI GER---MSSH YFQV 218

Clupea harengus_XP012689768.1 127 TFIQTDKPIYS PGQTVHYRVV TMDTDFIPLH QKYNLVSLQD SRGNTIGQWL NVTSQSKMVQ LSHVLNPEAL QGKYKLSVTR EQS---AFSH DFQV 218

Tenualosa ilisha 127 TFIQTDKPIYS PGQTVHFRVV TMDPDFIPLS EEYPHMSLQD SRGNVIGQWL NVRSQGKIVQ LSHVLNPEAP QGKYTLKING KQTSIQTRTH EFEV 218

* ******* ****** *** ** * * * * * ** * * * * * * * * *

**A2M_N_2 domain**

Homo sapiens_NP_000005.2 461 LEPMS HELPCGHTQT VQAHYILNGG 486

Danio rerio_NP01132951 461 IEDIE QPLKCGTEIT ATVKYYFVG- 485

Clupea harengus_XP012689768.1 467 IKAID EPLQCDKDTP ISIKYTFTG- 491

Tenualosa ilisha 472 IKPLD DDLPCGKDVA ITTKYVFIG- 496

* * * *

Homo sapiens(NP_000005.2) 487 TLLGLKKLSF YYLIMAKGGI VRTGTHGLLV KQE------D MKGHFSISIP VKSDIAPVAR LLIYAVLPTG DVIGDSAKYD VENCLANKVD LSFSPSQSLP 581

Danio rerio_NP01132951 486 ETVEDFSTDI VYMVLSRGVI VHHGFEKVEV KSS---SNGL ASGTMSFKLS VGADVAPVVQ ILAYCVLPSE NVIAANKRLD TEKCFGNKVS LQFSPAKAVP 583

Clupea harengus_XP012689768.1 492 ETFNTDSVDI IYMVLSKGEI VHHGFTEASV KASGKGSQPV ITGEVSFKLP VNPGMAPSVQ VLAYCVLPSE TVLAESRTFS TEKCFRNKVL VQFSPPKAVP 592

Tenualosa ilisha 497 ETFNTDHVDI VYMALSKGEI VHHGFTEPPV QGS----QPV TEGEVSFQVP IRAEMAPSVQ VLVYCILPSE TVLADSRTFS TEKCFKNKVA LQFSPPKAVP 591

* * * * * * * * * ** ** * * *** *** *

Homo sapiens_NP_000005.2 582 ASHAHLRVTA APQSVCALRA VDQSVLLMKP 607

Danio rerio_NP01132951 584 GEKNTLQLSA QPGSLCGLSA VDQSVLILES 611

Clupea harengus_XP012689768.1 593 GEKSSLQLSA QPGSLCGLTA VDQSILILEP 618

Tenualosa ilisha 592 GEQNSLQISA QPGSLCALSA VDQSIFILEP 619

* ** * * * * ****

**A2M domain**

Homo sapiens_NP_000005.2 737 TWIWDLV VVNSAGVAEV GVTVPDTITE WKAGAFCLSE DAGLGISSTA SLRAFQPFFV ELTMPYSVIR GEAFTLKATV LNYLPKCIRV SVQL 828

Danio rerio_NP01132951 729 TWIWELA EVGDSGSAEV PVTVPDTITT WETEAFCLSS TG-LGLAPPA QLTVFQPFFL ELSLPYSIIR GEIFELKATV FNYLSKCIMV KVSP 818

Clupea harengus_XP012689768.1 739 TWIWDLA EVGESGEAQV PLTVPDTITT WETEAFCLSP QG-FGLAPPV QLTVFQPFFL ELSLPYSIIR GETFELKATV FNYLPKCIMV TATP 831

Tenualosa ilisha 746 TWIWDLA EVGVSGEAQL PLTVPDTITT WETEAFCLSP QG-FGLAPPV QLTVFQPFFL ELSLPYSIIR GETFQLKATV FNYLPNCIMV TVTP 838

**** * * * * ******* * ***** * ***** ** ** ** ** ** ***** *** *

**A2M_2 and A2M_Complement domain & Thioester motif**

Homo sapiens_NP_000005.2 958 AMQNTQN LLQMPYG**CGE** 975

**Thioester**

**motif**

**(CGE)**

Danio rerio_NP01132951 948 ALQNLHG LLRMPYG**CGE** 965

Clupea harengus_XP012689768.1 961 ALKNIDS LLKMPYG**CGE** 978

Tenualosa ilisha 968 ALKNIDS LLKMPYG**CGE** 985

* * ** *******

Homo sapiens_NP_000005.2 976 QNMVLFAPNI YVLDYLNETQ QLTPEIKSKA IGYLNTGYQR QLNYKHYDGS YSTFGERYGR NQGNTWLTAF VLKTFAQARA YIFIDEAHIT QALIWLSQRQ 1076

Danio rerio_NP01132951 966 QNMAVLSPNI YILQYLENTK QLTSAIREKA SSFLKSGYQR QLNYKHFDGA YSTFGY---- GDGNTWLTAF VLRSFGKAQK YTFIDPQIIQ SAKDWLISRR 1062

Clupea harengus_XP012689768.1 979 QNMAILSPNI YILQYLQNTN QLTADIRERA THFLKSGYQR QLNYKHRDGA YSTFGR---- GEGNTWLTAF VLRSFGKAKS LIYIDPQIIT DAKTWLEGRL 1075

Tenualosa ilisha 986 QNMAILSPNI YILQYLQNTG QLTAEIREKA TNFLKNGYQR QLNYKHFNGA YSTFGS---- GEENTWLTAF VLRSFGKAKS FVYIDPVIIE SAKTWLLTKL 1082

*** *** * * ** * *** * ** * **** ****** * ***** ******* ** * ** * * **

Homo sapiens_NP_000005.2 1077 KDNGCFRSSG SLLNNAIKGG VEDEVTLSAY ITIALLEIPL TVTHPVVRNA LFCLES---- ---------- AWKTAQEGDH GSHVYTKALL AYAFALAGNQ 1163

Danio rerio_NP01132951 1063 DSDGCFIQQG RLFNNRMKGG VNDNVTMTAY ITASLLELET PVTDPVITKG LS-------- ---------- --CLRSVIEE VKNTYTTALL AYTFSLARDT 1143

Clupea harengus_XP012689768 1076 RPDGGFHMQG KLFNNRMKGG VNDNVTITAY ITASMLELNM SVTEKTLTFL KS-------- ---------- ------SVSD LSNTYSTALL AYTFSLAGEE 1150

Tenualosa ilisha 1083 QPDGAFFMQG KLFNNRMKGG VSDDLTISAY IAATLLELNM TVPEHTMAFL KSGYSGSPPP PRDLSHAREI LMFLRPSTND FSNTYTTALL AYTFSLAGEE 1183

**A2M Complement**

* * ** **** * * * ** ** * * ** **** **

Homo sapiens_NP_000005.2 1164 DKRKEVLKSL NEEAVKKDNS VHWERPQKPK APVGHFYEPQ APSAEVEMTS YVLLAYLTAQ PAPTSEDLTS ATNIVKWITK QQNAQGGFSS TQDTVVALHALSKY 1264

Danio rerio_NP01132951 1144 NTRQQLFNKL EDLAISDGPL VHWSR----- ----SASADD SASLDVEISS YVLLAVLTAD -SLTTADLGF ANRIVSWLVK QQNAYGGFSS TQDTVVALQALSLY 1221

Clupea harengus_XP012689768 1151 DLRSQLLKHL DSVATSDGNL LHWS------ ----HSSSER ADSLAVETSS YVLLAVFSKT -TLTAADLGY AARIVNWLVK QQNPYGGFSS TQDTVVALQALSLY 1241

Tenualosa ilisha 1184 DIRAQLLKHL DSVATSSGNL LHWS------ ----QSSSER ADSLAVETSS YVLLAVLTKS -TLTAADLGY AARIVSWLVK QQNPYGGFSS TQDTVVALQALSLY 1272

* ** * ***** ** ** ** * * *** ***** ******** *** *

**Bait Region**

Homo sapiens(NP_000005.2) 690 ---PQLQQYEMHG----PEGLRVGFYESDVMGRGHARLVHVEEPHT- 728

Danio rerio_NP01132951 679 ---LSYRGLTYHKNLVMYPDHRPVPVMFALGMAGVDGPVGNSPS--- 719

Clupea harengus_XP012689768.1 694 ---LLYRGDTYHK------GHAYPEVAYGVAFNGMAGPGGPPPEESG 732

Tenualosa ilisha 695 LTFLGRRYYLNHGYRVIQHEHGYMVLSSSGVGGGMFGPSREEFG--- 739

: *

**A2M receptor**

Homo sapiens_NP_000005.2 1374 SASNM AIVDVKMVSG FIPLKP--TV KMLERSNHVS RTEVSSNHVL IYLDKVSN-Q TLSLFFTVLQ DVPVRDLKPA IVKVYDYYETD 1456

**A2M receptor**

Danio rerio_NP01132951 1339 EKTNM AIVDIKLLSG FTADTSALGT SSGTYVSLVE RVDSKDDHVI VYLKEISKNV AMNYQIQMKQ VLQVKNLKPA VVKVYDYYQTS 1414

Clupea harengus XP012689768.1 ----- ---------- ---------- ---------- ---------- ---------- ---------- ---------- -----------

Tenualosa ilisha 1353 QSTNM AIIDVTMLSG FTPDSGTVER --LQSSKYVD RVDKKDDHIL VYLSALPKAM SIHYLLIIQQ DLLVNNLKPA VVKVYDYYQTS 1465

** **** ** * * * * ** * * **** ******* *

**Figure** B. Domain Identification in Hilsa A2ML protein (consensus protein) in reference to *Homo sapiens, Danio rerio* and *Clupea harengus*. Multiple sequence alignment of *T. ilisha* A2ML protein with representative sequences shows highest similarity in A2M_N, A2M, A2M_2, A2M_complement and A2M_receptor domain. Little similarity was found in Bait region.

Homo_NP000005.2 SISIPVKSDIAPVARLLIYAVLPTGDVIGDSAKYDVENCLANKVDLSFSPSQSLPASHAH 584

Cyprinus_ABC47740.2 SFKLSVGADLAPAVQILAYCVLPSENVATGSAKFDVEKCFSNKVSLQFSPAKAVPGEKNT 60

Liver_20636 SFQVPIRAEMAPSVQVLVYCILPSETVLADSRTFSTEKCFKNKVALQFSPPKAVPGEQNS 596

Liver_19153 SFQVPIRAEMAPSVEVLVYCFSPSETVLADSRTFSTEMCFKNKVVLHFSPPKAVPGEQNS 132

Testes_18316 ------------------------------------------------------------ 0

Ovary_19791 ------------------------------------------------------------ 0

Homo_NP000005.2 LRVTAAPQSVCALRAVDQSVLLMKPDAELSASSVYNLLPEKDLTGFPGPLNDQDDEDCIN 644

Cyprinus_ABC47740.2 LQLSAQPGSLCGLSAVDQSVLIMESGKRLDTEKIFNLLPVQSVSGYPQGVED--EQECLH 118

Liver_20636 LQISAQPGSLCALSAVDQSIFILEPGKRLDVQKVFGLLPELNS-EVHYDAED--HVECFK 653

Liver_19153 LQISAHPGSLCGLSAVDQSIFILEPRKRLDAQKVFGLLPVLNS-EVHYDVED--HVECTE 189

Testes_18316 ------------------------------------------------------------ 0

Ovary_19791 ------------------------------------------------------------ 0

Homo_NP000005.2 RHNVYINGITYTPVSSTNEKDMYSFLEDMGLKAFTNSKIRKPKMCPQLQQYEMHGPEGLR 704

Cyprinus_ABC47740.2 V**RSRR**ALSTD----------NAFESLKRVGLKMATNLAVPIPQ-CLSYRGLTYFRYQVFH 167

Liver_20636 V**RPRRS**SMPYHYPRH--SDSGVSKVFKILGLKTISNLALQIPD-CLTFLGRRYYLNHGYR 710

Liver_19153 V**RPRR**SYVLDYHPWH--SDTGVSAVFKRLGLKIVSNFA-QIPN-CLIFKGWRYHRHDGFY 245

Testes_18316 ------------------------------------------------------------ 0

Ovary_19791 ------------------------------------------------------------ 0

Homo_NP000005.2 VG-----FYESDVMGRGHARLVHVEEPHTETVRKYFPETWIWDLVVVNSAGVAEVGVTVP 759

Cyprinus_ABC47740.2 PPVSMPV-AVMY-SLADGDFGTSSRDSPAVTIRTVFPETWIWELAEVGDSGSTQVPVTVP 225

Liver_20636 VIQHEHGYMVLSSSGVGGGMFGPSREEFGVTVRTVFPETWIWDLAEVGVSGEAQLPLTVP 770

Liver_19153 GERD---L-VM-------HRSHESSKKFGVTVRTVFPETWIWDLAEVGESGEAQLPLTVP 294

Testes_18316 ----------M-------HRSHESSKKFGVTVRTVFPETWIWDLAEVGESGEAQLPLTVP 43

Ovary_19791 ----------M-------HRSHESSKKFGVTVRTVFPETWIWDLAEVGESGEAQLPLTVP 43

. *:*. *******:*. *. :* ::: :***

Homo_NP000005.2 DTITEWKAGAFCLSEDAGLGISSTASLRAFQPFFVELTMPYSVIRGEAFTLKATVLNYLP 819

Cyprinus_ABC47740.2 DTITSWETEAFCLSS-KGLGLAPPAQLTVFQPFFLELSPPYSIIRGEIFELKATVFNYLS 284

Liver_20636 DTITTWETEAFCLSP-QGFGLAPPVQLTVFQPFFLELSLPYSIIRGETFQLKATVFNYLP 829

Liver_19153 DTITTWETEAFCLSP-QGFGLAPPVKLTVFQPFFLELSLPYSIIRGETFELKATVFNYLP 353

Testes_18316 DTITTWETEAFCLSP-QGFGLAPPVKLTVFQPFFLELSLPYSIIRGETFELKATVFNYLP 102

Ovary_19791 DTITTWETEAFCLSP-QGFGLAPPVKLTVFQPFFLELSLPYSIIRGETFELKATVFNYLP 102

**** *:: ***** *:*:: ..* .*****:**: ***:**** * *****:***

Homo_NP000005.2 KCIRVSVQLEASPAFLAVPVEKEQAPHCICANGRQTVSWAVTPKSLGNVNFTVSAEALES 879

Cyprinus_ABC47740.2 KCIMVKVTPAPSSDYTLKASADDQYSSCLCANGRKTFKWILTPSVLGVMNITVSAEAEAS 344

Liver_20636 NCIMVTVTPAPSSDFTLTPSYDGQYSSCLCAHERKTFTWTLVPSVLGLMNVTVSAKASHS 889

Liver_19153 NCIMVTVTPAPSSDFTLTPSSDGQYSSCLCAHGRKTFTWTLVPSVLGLMNVTVSAKASHS 413

Testes_18316 NCIMVTVTPAPSSDFTLTPSSDGQYSSCLCAHGRKTFTWTLVPSVLGLMNVTVSAKASHS 162

Ovary_19791 NCIMVTVTPAPSSDFTLTPSSDGQYSSCLCAHGRKTFTWTLVPSVLGLMNVTVSAKASHS 162

:** *.* * : . * *:**: *:*..* :.*. ** :*.****:* *

Homo_NP000005.2 QELCGTEVPSVPEHGRKDTVIKPLLVEPEGLEKETTFNSLLCPSGGEVSEELSLKLPPNV 939

Cyprinus_ABC47740.2 QTVCDNEIVSVPERGHIDTVTRSLLVQAEGTEKTETYSWLLCPKGDSLSEEVNLTLPKDV 404

Liver_20636 EALCGNEVVSVPERGRIDIVTRPLLVKAEGTEKTESFNWLLCPKGGALTEEVELKLPQNV 949

Liver_19153 EALCGNEVVSVPERGHIDIVTRPLLVKAEGAEKSASFNWLLCPKGGALTEEVELKLPAAV 473

Testes_18316 EALCGNEVVSVPERGHIDIVTRPLLVKAEGAEKSASFNWLLCPKGGALTEEVELKLPAAV 222

Ovary_19791 EALCGNEVVSVPERGHIDIVTRPLLVKAEGAEKSASFNWLLCPKGGALTEEVELKLPAAV 222

: :*..*: ****:*: * * : ***: ** ** ::. ****.*. ::**:.*.** *

Homo_NP000005.2 VEESARASVSVLGDILGSAMQNTQNLLQMPYGCGEQNMVLFAPNIYVLDYLNETQQLTPE 999

Cyprinus_ABC47740.2 IEGSARSSVSVIGDILGRALRNLNGLLRMPYGCGEQNMAVLSPNIYILQYLENTEQLTSA 464

Liver_20636 VQGSARASVSVLGDILGRALKNIDSLLKMPYGCGEQNMAILSPNIYILQYLQNTGQLTAE 1009

Liver_19153 VQGSARASVSVLGDILGRALKNIDSLLKMPYGCGEQNMAILSPNIYILQYLQNTGQLTAE 533

Testes_18316 VQGSARASVSVLGDILGRALKNIDSLLKMPYGCGEQNMAILSPNIYILQYLQNTGQLTAE 282

Ovary_19791 VQGSARASVSVLGDILGRALKNIDSLLKMPYGCGEQNMAILSPNIYILQYLQNTGQLTAE 282

:: ***:****:***** *::* :.**:**********.:::****:*:**::* ***

**Figure C.** Alignment of Bait region (deduced amino acid sequences) of *T. ilisha* isoforms (four isoforms having bait regions, Liver_20636, Liver_19153, Testes_18316 and Ovary_19791) with *Homo sapiens* (NP000005.2) and *Cyprinus carpio* (ABC47740.2) are shown in shaded region. The beta–alpha processing signal (RSRR in *C. carpio* and RPRR in *T. ilisha* Liver isoforms) is underlined.

**Marker 1**

Cyprinus_carpio_BAA85038 191 ELNPEARQGVYRLKTYIGER---M***I***SHDFEVKKYVLPKYEVTVKRPNEV 237

Oreochromis_niloticus_XP019212 188 SLDTEAREGPYQIIVSMGET---K***I***SHNFKVEKYVLPKFDVTVNISEEV 234

Maylandia_zebra_XP014269643 188 SLDAEAPEGPYQIIVSVGER---K***I***SHNFKVEKYVLPKFDVTVNISEEV 234

Takifugu_rubripes_XP011616382 192 PMIPEAEQGRYTIGAKTDKG— ER***I***LHGFDIKEYVLPKYEVKVHLPQVI 239

Poecilia_X1_XP008423358 192 ASIPEAAQGTYTISVVTDQE— EE***I***SHTFDIKEYVLPKFEVKVDLPSVI 239

Labrus_XP020512318 194 PMNSDAEQGTYTISAITDKG— EK***I***THTFVVKEFVPPKYEVTVQLPSMV 241

Larimichthys_X1_XP019131243 194 PMIPEAPQGSYIITASTDKG— ER***I***THSFDIKEYVLPKYEVKIHLPSVI 241

Fundulus_XP021166416 193 LSVPEAAQGTYTISAETDVG— EE***I***SHTFDIKEYVLPKFEVKVDLPSVI 240

Monopterus_XP020476381 190 PLIPEAPQGRYTITASTDKG— EQ***I***SHSFDVKEYVLPKYEVKVHLPSVI 239

Clupea_XP012689768 191 VLNPEALQGKYKLSVTREQS---A***F***SHDFQVKKYVLPKFEVIPNIPEEV 237

Danio rerio_NP01132951 191 ELNPECREGAYKLKAFIGER---M***S***SHYFQVKKYVLPKFEVTVKKPKTV 237

Ctenopharyngodon_idella_AAR003 ---------------------------------YVLPKFEVTMKAPKAI 16

Salmo_salar_XP014069237 192 PMSAEATQGSYIITAWNEKG— EQ***T***SQNFDVKEYVLPKYEVKVYLPQTI 239

Rachycentron_canadum_AIT68782 191 NLNSEAHEGYYQVIVSFGEK---K***V***YHSFKVEKYVLPKFDVTLNVLDEV 237

Carassius_gibelio_AGU16534 201 AIPEMASPGIWKVVTLFSNTPQKK***Y***TADFEVKEYVLPAFEVKLKLSKSF 250

Esox_X1_XP010891963 191 SLNSEAREGSYLISAWVGEN---A***R***SHMFTVEKYVLPKFEIKITHDEI- 236

Tenualosa ilisha 194 VLNPEAPQGKYTLKINGKQTSIQT***R***THEFEVKKYVLPKFDVTLTTPKDI 243

:* * ::: .

**Marker 2**

Cyprinus_carpio_BAA85038 258 PGKSWVKVCRN***I***LPY------------LQARDKRNPLCLEETTEIKKTGC 296

Oreochromis_niloticus_XP019212 255 PGRVTVNVCRP***I***NWYSGISIGLYDY-DLLALLQMRGSCRIETKQADKTGC 305

Maylandia_zebra_XP014269643 255 PGRVTVNVCRP***I***NLYRGFGILVHSE-DDLALLQISAPCHTETKQADKTGC 305

Takifugu_rubripes_XP011616382 259 IGSVKADFCRS***A***FAFYWY-----------SGEQPKDICKTYQLTTDKSGC 298

Poecilia_X1_XP008423358 259 LGRVKAEFCKK***Y***FLFFWR-----------SEEPDNDICKSFDLTLDKSGC 298

Labrus_XP020512318 260 QGSIKAVFCR-***I***GSVYWY-----------YRTKDINPCKTYNMTTDKSGC 299

Larimichthys_X1_XP019131243 259 IGSVKAVFCRH***A***LRFYWY-----------STSEENDVCSVYELTTDKSGC 298

Fundulus_XP021166416 260 IGTVKAEFCRK***S***FVFFWR-----------TEEPNNDICKSYDLTLDKTGC 299

Monopterus_XP020476381 257 LGAVKAVVCRN***A***FQFYWN-----------SHLSENDICRNYIMTTEKSGC 296

Clupea_XP012689768 258 PGKASMVLCRD***A***DVNQFE-------- EDDGAAL—EICLKEQGEMDESGC 298

Danio rerio_NP01132951 258 PGKSWVKVCRN***P***LPY------------IIFF----PLCLEETLEITKTGC 292

Ctenopharyngodon_idella_AAR003 38 AGKARVEVCRE***L***LKY------------ISRPD-LISPCLVKTIEISKVGC 75

Salmo_salar_XP014069237 259 MGSVTATVCRI***A***VQYHWL-----------YDSS— NICEVYVIKTDKTGC 296

Rachycentron_canadum_AIT68782 257 PGSVEIDVCRL***H***NHYDMMLPTITLNPEGVTQIFEINPCHKETKQTDGKGC 307

Carassius_gibelio_AGU16534 271 DGNAFVVFGVM***G***DEK--------------KISIPASLQKVQIMKGEGTAE 307

Esox_X1_XP010891963 257 PGKAELDLCRP***F***GYNMHI-------- LFMEDLKP—PCVKQSVELDQTGC 297

Tenualosa ilisha 261 PGKADLSLCRD***V***KPYRDY--------EDESTAEPQAPCITVSVWMDGSGC 303

* . .

**Marker 3**

Cyprinus_carpio_BAA85038 462 ELIIENIEQPLKCD***A***EFTVTIKYYFIGETVE—DFKTDIVYMVLSRGLI 509

Oreochromis_niloticus_XP019212 467 SLEVQKKDKSVPCD***A***EEDISVNYTIVGESP----GSVDVIYLVLSRGAV 512

Maylandia_zebra_XP014269643 468 SLEVQKKDKSLPCD***A***EEDVSVNYTIVGESP----GSVDVIYLVLSRGAV 513

Takifugu_rubripes_XP011616382 440 FLKLTQGNGKLSCG***Q***EASVRVQYIIQGEELRKGQEVLDHFYLVMSKGRI 489

Poecilia_X1_XP008423358 456 FLMLKHISDGFACD***R***DATVTAQYIIQGTELKEGQTVLNFFYLVLSKGET 505

Labrus_XP020512318 454 FLKIMLADGKISCD***R***DATVHAQYIIQGKALRYETDTLTFFYLVMSKGVM 503

Larimichthys_X1_XP019131243 453 FLKLMQIKGKLSCD***S***DATVRAQYIIQGEELKKGQEVLEFFYLVMSRGGI 502

Fundulus_XP021166416 457 FLKLKQISEGLPCD***R***DATVKAQYIIQGKELKEGQTVLNFFYLVLSKGAI 506

Monopterus_XP020476381 454 FLKLMQVSEKISCD***K***DVAVRAQYIIQGQELKMGQEVLDFFYLVMSRGDI 503

Clupea_XP012689768 464 SVTIKAIDEPLQCD***K***DTPISIKYTFTGETFN—TDSVDIIYMVLSKGEI 511

Danio rerio_NP01132951 458 ELIIEDIEQPLKCG***T***EITATVKYYFVGETVE—DFSTDIVYMVLSRGVI 506

Ctenopharyngodon_idella_AAR003 241 ELIIENTEQPLKCD***S***EFTATIKYYFVGETVE—DFKTDIVYMVLSRGVI 288

Salmo_salar_XP014069237 456 FVKIMQGEGKFSCE***K***DGIVLARYIIHGVELKRGQTTLDFFYLVISRGSI 505

Rachycentron_canadum_AIT68782 472 TLEVKKNKEAFPCG***Q***EQDISIQYTVVGEEQ----GSAHVMYLVLSRGTI 517

Carassius_gibelio_AGU16534 457 YLHIGIDAAELQIG***D***PIKVNLN---TGQSPG—VKDQDLTYMILSKGQI 501

Esox_X1_XP010891963 462 SLSVQKIEEPMPCG***E***EVSITVQYAIVGETVP—KGSVDVIYLALSRGVL 509

Tenualosa ilisha 469 SLTIKPLDDDLPCG***K***DVAITTKYVFIGETFN—TDHVDIVYMALSKGEI 516

: : . . * *: :*:*

**Marker 4**

Cyprinus_carpio_BAA85038 558 AGSTQFDVEKCF***S***NKVSLQFSPAKAVP--GEKNTLQLSAQPGSLCGLSAVD 606

Oreochromis_niloticus_XP019212 560 AHSADFSTDKCF***S***NKVSVEFSPSSAVP—GEETNMQVTALPRSLCGVSAID 608

Maylandia_zebra_XP014269643 561 AHSADFSTDKCF***S***NKVSVEFSPSSAVP—GEETNMQVTALPRSLCGVSAID 609

Takifugu_rubripes_XP011616382 552 ADSQDFPVRLCL***N***NKVSLKFSSLQELP—AEKTTLSLKAHPGSLCSVRAID 600

Poecilia_X1_XP008423358 553 ADSQDFPIQLCL***S***NKVSLKFSSVQQLP—AEQTTLTLKANPKSLCSVRAID 601

Labrus_XP020512318 551 ADSMNFPVELCF***N***NKVSLKFSSVQQLP—AEETKLQLNAEPGSMCSVRAID 599

Larimichthys_X1_XP019131243 550 ADSQDFPIQLCL***S***NKVSLKFSSLQELP—AEKTTLNLQAHPGSMCSVRAID 598

Fundulus_XP021166416 554 ADSQDFPVQLCL***N***NKVSLKFSSVQKLP—AEEVTLTLKANPKSLCSVRAID 602

Monopterus_XP020476381 551 ADSQDFPIQLCL***N***NKVSLQFSSLQELP—AEETTLRLQAHPGSVCSVRAID 599

Clupea_XP012689768 564 AESRTFSTEKCF***R***NKVLVQFSPPKAVP—GEKSSLQLSAQPGSLCGLTAVD 612

Danio rerio_NP01132951 555 AANKRLDTEKCF***G***NKVSLQFSPAKAVP—GEKNTLQLSAQPGSLCGLSAVD 603

Ctenopharyngodon_idella_AAR003 337 AGSKNFEIEKCF***K***NKVSLQFSPDKAVP—GEKNTLQLSAQPGSLCGLSAVD 385

Salmo_salar_XP014069237 553 ADSQDFPIQLCL***K***NKVSLKFSSLQELP—GEKTSLSLQAHPGSLCSLRAID 601

Rachycentron_canadum_AIT68782 565 ANSATFTTEKCF***G***HKVSLEFSPSSAVP—GEETTMQVTAEPDSLCGVSAVD 613

Carassius_gibelio_AGU16534 544 SDSVWVDVKDTC***M***GKLQIKVKDKMNTYGTGDEVKLQITGDPGAKVGLVVVD 599

Esox_X1_XP010891963 558 AHSTKFPIEKCF***R***NKVSVEFSPSKAVP—GEKNTLQLSAQPGSLCGLSAVD 606

Tenualosa ilisha 559 ADSRTFSTEKCF***K***NKVALQFSPPKAVP—GEQNSLQISAQPGSLCALSAVD 607

: . . *: ::.. .:: .: : . * : .: .:*

**Marker 5**

Cyprinus_carpio_BAA85038 900 LLVQAEGTEKTET***Y***SWLLCPKVDSLSEEVDLNLPKDVIEGSARSSVS 947

Oreochromis_niloticus_XP019212 909 LIVKAEGTEMTKT***Y***NWLLCPKGSPLTEEAEIHLPENVIEGSARTSVS 956

Takifugu_rubripes_XP011616382 908 LLVEAEGTPQMVS***H***NALLCPAGGPKEKKVSLLLPEFFVAGSARASVS 955

Poecilia_X1_XP008423358 922 LLVEAEGTPQMKS***H***NTLLCPADGPVEKKISLQRPEMFVEGSVRASVS 969

Labrus_XP020512318 926 LLVEAEGTPQMVS***H***SALLCPAKETVEENMSLELPKDYVEGSVRASVS 973

Larimichthys_X1_XP019131243 940 LLVEAEGTPQMVS***H***NALLCPAEGPVERKISLLMPEMFVAGSARASVS 987

Fundulus_XP021166416 958 LLVEAEGTPQMES***H***NALLCPGNSPVEKKISLLLPAAFVAGSARASVS 1005

Monopterus_XP020476381 895 LLVEAEGTPQMVS***H***NALLCPAEGPVEKNISLLMPEMFVAGSARASVS 942

Clupea_XP012689768 906 LRVKAEGTEKTES***F***SWLLCPKGGALTEEVELKLPEAVVKGSSRASVS 953

Danio rerio_NP01132951 893 LLVQAEGIEKTET***N***SWLLCPKGDSLSEKVDLTLPKDVIEGSAKSSVS 940

Ctenopharyngodon_idella_AAR003 665 LLVQAEGTEKTET***H***SWLLCPKGDSLSEEVALTLPKDVIEGSARSTVS 712

Salmo_salar_XP014069237 916 LLVEAEGTQETVS***H***NALLCPAEGPVEKDISLKLPEVFVEGSAKASLS 963

Rachycentron_canadum_AIT68782 915 LIVKAEGTEMTKT***H***NWLLCPKGETLTEEIELQLPNDVIDGSARASIS 962

Carassius_gibelio_AGU16534 923 HRKNVELNPVKNG***E***KPIVVKSEIPVDR-VPDTPANTYISITGEEIAQ 970

Esox_X1_XP010891963 910 LLVKAEGTEKTDT***H***NWLLCPTGEALTKEVELQLPKNVVDGSDRASLS 957

Tenualosa ilisha 913 LLVKAEGTEKTES***F***NWLLCPKGGALTEEVELKLPQNVVQGSARASVS 960

:.* . :: . . . : : . .

**Figure D.** Putative markers of EUS disease in fishes. Multiple sequence alignment of A2ML protein in EUS resistant (*Cyprinus carpio, Oreochromis niloticus and Maylandia zebra* , EUS susceptible fishes (Green shaded) are shown where fixed pattern of mutations identified at five positions in resistant fishes.
